# Magnesium supplementation beneficially affects depression in adults with depressive disorder: a systematic review and meta-analysis of randomized clinical trials

**DOI:** 10.3389/fpsyt.2023.1333261

**Published:** 2023-12-22

**Authors:** Mahdi Moabedi, Mohammadreza Aliakbari, Shima Erfanian, Alireza Milajerdi

**Affiliations:** ^1^Student Research Committee, Kashan University of Medical Sciences, Kashan, Iran; ^2^Student Research Committee, Isfahan University of Medical Sciences, Isfahan, Iran; ^3^Research Center for Biochemistry and Nutrition in Metabolic Diseases, Institute for Basic Sciences, Kashan University of Medical Sciences, Kashan, Iran

**Keywords:** magnesium, depressive disorder, adults, meta-analysis, clinical trials

## Abstract

**Background:**

The findings from randomized clinical trials (RCTs) examining the effect of magnesium supplementation on depression are inconsistent. We decided to conduct a meta-analysis that summarizes all the evidence on the impact of magnesium supplementation on depression scores in adults with depressive disorder.

**Methods:**

We conducted a systematic search in the online databases using all related keywords up to July 2023. We included all randomized clinical trials examining the effect of magnesium, in contrast to placebo, on depression scores.

**Results:**

Finally, seven clinical trials were included in this systematic review, building up a total sample size of 325 individuals with ages ranging from 20 to 60 years on average. These RCTs resulted in eight effect sizes. Our findings from the meta-analysis showed a significant decline in depression scores due to intervention with magnesium supplements [standardized mean difference (SMD): −0.919, 95% CI: −1.443 to −0.396, *p* = 0.001].

**Conclusion:**

Our review suggests that magnesium supplementation can have a beneficial effect on depression. Future high-quality RCTs with larger sample sizes must be run to interpret this effect of magnesium on depression in clinical settings.

**Systematic review registration:**

https://www.crd.york.ac.uk/prospero/display_record.php?RecordID=447909.

## Introduction

Depression is defined as a mental disorder characterized by sadness, loss of interest or pleasure, feelings of guilt or low self-worth, disturbed sleep or appetite, feelings of tiredness, and poor concentration (1). It is a common, debilitating, and potentially lethal disorder (2). It causes negative socioeconomic influences due to functional disabilities (3). Approximately 3.8% of the population experiences depression, including 5% of adults and 5.7% of the elderly (1).

Antidepressant medications and/or psychotherapy are effective in improving some symptoms of depression. However, more than half of all patients who used these medications reported side effects (4). Therefore, several complementary therapies, such as vitamin and mineral supplementations, have been looked into in depressed individuals. It is believed that administration of these supplements can reduce symptoms of depression with minimal side effects.

Magnesium acts as a co-factor in more than 350 enzymes in humans; the majority of them play a crucial role in brain function. It affects mood regulation by balancing chemical compounds in the brain (5). Magnesium increases the expression of brain-derived neurotrophic factor (BDNF), through which it may reduce the function of the N-methyl-D-aspartic acid (NMDA) ionotropic glutamate receptor. Magnesium is considered a potent antagonist of the NMDA receptor complex, while it is an inhibitor of glycogen synthase kinase 3 (GSK-3), similar to several well-known antidepressants (6). Furthermore, several studies suggest that the serum concentration of magnesium might be changed during depressive episodes. A study by Styczeń et al. (7) showed that average serum levels of magnesium were higher in patients with depressive episodes than in healthy volunteers. Another study by Wildmer et al. (8) showed similar results. In contrast, Dominguez et al. (9) suggested an inverse association between serum magnesium and depression. Moreover, findings from clinical trials investigating the effects of magnesium supplementation on depression are inconsistent. A study by Afsharfar et al. (10) showed that intervention with 500 mg/day of magnesium over 8 weeks improved depression symptoms (3.53 ± 4.63 points reduction in DBI score). In contrast, some other studies, such as Ryszewska-Pokraśniewicz et al. (11), did not find a significant effect of magnesium supplementation in the reduction of depression scores in comparison with magnesium and fluoxetine or fluoxetine alone after 8 weeks of intervention.

Regarding controversial findings from RCTs on the effects of magnesium supplementation on symptoms of depression, we decided to conduct a systematic review and meta-analysis to summarize results from RCTs on the impact of magnesium supplementation on depression in adults with depressive disorder.

## Methods

The study protocol is available at PROSPERO (CRD42023447909). This study was performed based on the PRISMA protocol for reporting systematic reviews and meta-analyses.

### Search strategy

We did a systematic search in the online databases of PubMed, Scopus, Web of Science, and Google Scholar up to July 2023, using the following keywords: [(“Magnesium” OR “mg”) AND (“major depression” OR “refractory depression” OR “depression scores” OR “affective disorders” OR “depressive disorder” OR “mental health” OR “depression”) AND (“RCT” OR “Randomized controlled trial” OR “Randomized clinical trial” OR “Random allocation” OR “Random assignment” OR trial OR trials OR randomized OR randomized OR controlled OR blind OR blinded OR cross-over); Supplementary Table 1].

No restrictions were made on the time or language of publications. In addition, we reviewed the citation list of the relevant articles to avoid missing any publication. Duplicate citations were also removed.

### Inclusion criteria

All the studies with the following criteria were included: (1) randomized controlled clinical trials, (2) studies performed on adults with depression, (3) studies that operated magnesium supplements in different forms, including magnesium sulfate, magnesium chloride, magnesium oxide, and magnesium aspartate, (4) clinical trials with a minimum intervention duration of 1 week, and (5) controlled trials that reported mean changes and their standard deviations (SDs) of depression scores throughout the trial for both intervention and control groups or required information for calculation of those effect sizes. If more than one article was published for one dataset, we included only the most complete one.

### Exclusion criteria

We excluded observational studies, review articles, and ecological articles. In addition to that, we removed clinical trials that did not have a placebo or control group. In addition to that, we removed non-randomized trials and clinical trials that did not have a placebo or control group. We also excluded articles in which children or adolescents were included. Moreover, unpublished studies and gray pieces of literature were removed during the screening process.

### Data extraction

Two independent investigators extracted the following information from the included studies: first author’s name, publication year, individuals’ characteristics (mean age and sex), study design, sample size (control and intervention groups), type of magnesium supplement prescribed, the dosage of magnesium, duration of intervention, mean changes, and their SDs of depression score throughout the trial for the intervention and control groups, and the confounding variables adjusted for.

### Quality assessment

The risk of bias for each included study was assessed using the Cochrane quality assessment tool (12). This tool contained seven domains, including random sequence generation, allocation concealment, reporting bias, performance bias, detection bias, attrition bias, and other sources of bias. Each domain was given a “high risk” score if the study comprised methodological defects, a “low risk” score if it did not have any methodological flaws for that item, and an “unclear risk” score if there was not sufficient information to establish the risk. We considered the overall risk of bias following these criteria: (1) low if all the domains were marked as “low risk,” (2) moderate if one or more domains were marked as “unclear risk,” and (3) high if one or more domains were marked as “high risk.”

### Statistical analysis

We extracted mean changes and SDs of depression scores in the intervention and control groups to acquire standardized mean differences (SMDs). When studies did not report mean changes, we obtained them using final and baseline reports of that variable. We also converted standard errors (SEs), 95% confidence intervals (CIs), and interquartile ranges (IQRs) to SDs using a suitable formula from the method introduced by Hozo et al. (13). A random-effects model that takes between-study variations into account was used for final analyses as there was a high between-study heterogeneity. Between-study heterogeneity was determined using the *I*^2^ statistic and Cochrane’s Q-test. *I*^2^ values >50% were considered significant between-study heterogeneity.

To find probable sources of heterogeneity, we performed different subgroup analyses using the predefined variables, including the type of supplementation (oral vs. infusion), depression assessment test (BDI I/II vs. non-BDI), study location (Iran vs. non-Iran), severity of depression (mild to moderate vs. major), duration of the interventions (8 vs. <8 weeks), and magnesium dosage (≤250 vs. >250 mg/day). We used sensitivity analysis to detect each individual study’s overall effect size. We examined publication bias using Egger’s regression test. We conducted the meta-analysis with the Stata software, version 17 (StataCorp). We considered a *p* value of <0.05 as statistically significant.

## Results

**Figure 1:**
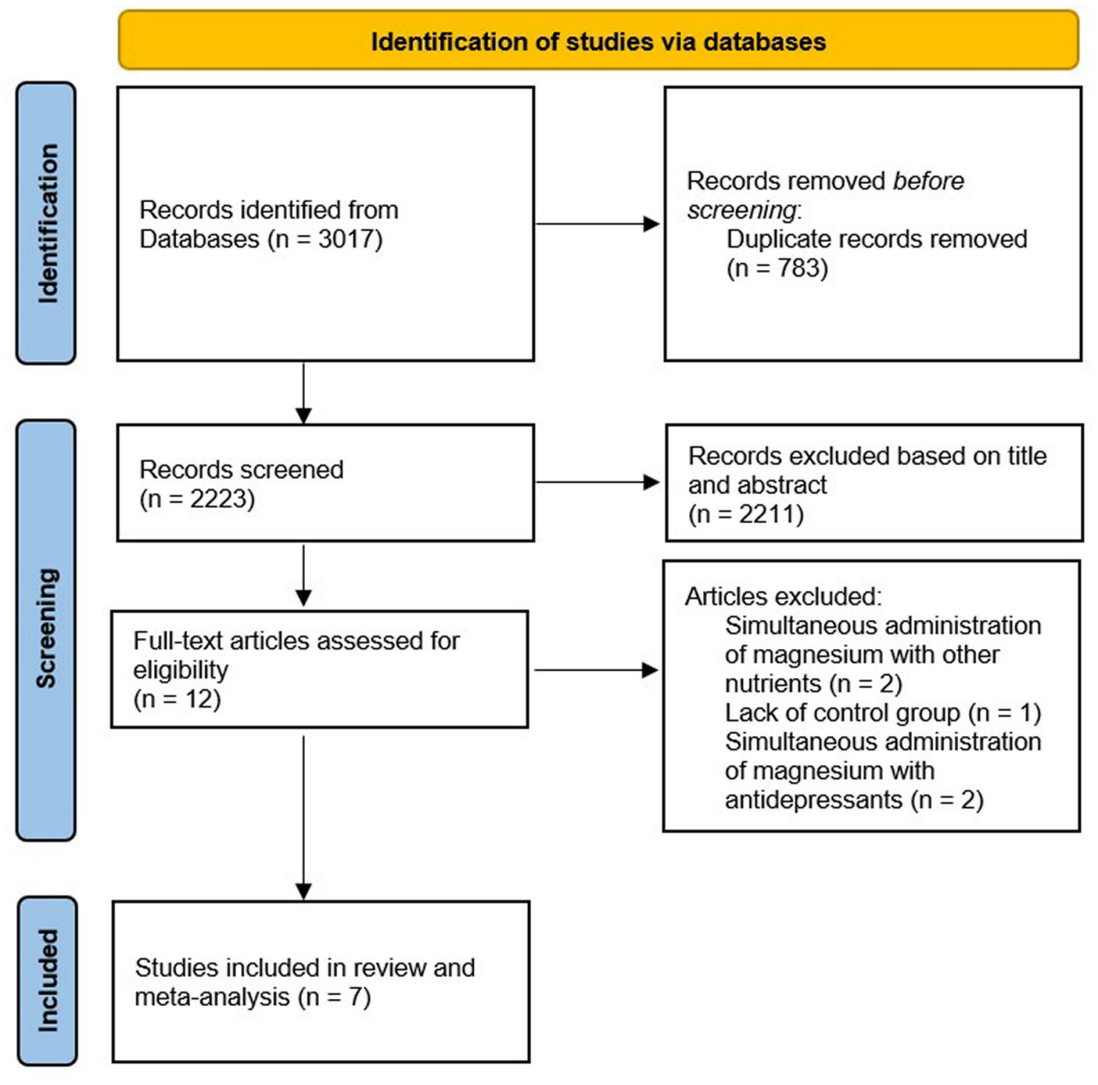
Flowchart of the study selection.

Overall, 3,017 publications were identified in our initial systematic search. We removed 783 duplicate articles. After skimming the remaining 2,223 records, considering title and abstract, 2,211 unrelated articles were excluded. Then, 12 publications remained for further evaluation. Out of those 12 studies, five articles were excluded by the full-text screening. Finally, seven eligible RCTs were included in the current systematic review and meta-analysis. Depression symptoms were assessed using *Beck’s Depression* Inventory (BDI) (5, 10), BDI II (14, 15), and the Hamilton Depression Rating Scale (HAM-D) (11, 16) among the included studies. Moreover, one study reported a mean depression symptom score (17). The flow diagram of study selection is shown in Figure 1.

### Study characteristics

The characteristics of the seven included RCTs are summarized in Table 1. These RCTs were published between 2016 and 2022 and were from the United States (16), Poland (11), and Iran (5, 10, 14, 15, 17). Two studies were exclusively performed on female subjects (14, 17), and the other five were performed on both genders. The sample size of our included RCTs was 12–66 individuals, which made up a total sample size of 325 adults. The studies had participants with a mean age of between 26.4 and 48.1 years. Included studies had interventions using 40–500 mg of magnesium daily of different types. The duration of the intervention varied from 1 to 8 weeks.

**Table 1:** Summary of clinical trials on the effects of magnesium supplementation on depression in adults aged ≥20 years.

Author, year	Participants, *n*	Design	Baseline depression	Age, year^1^	Depression test	Anti-depressant usage	Intervention		Duration (week)	Outcomes (score changes)	
							Treatment group	Control group		Treatment group	Control
Abiri et al. (18)	Fe: 51 Intervention: 26, Control: 25	R/DB/parallel	MI to MO	Intervention: 34 ± 9, Placebo: 34 ± 9	BDI II	No	250 mg/day magnesium oxide	Placebo	8	−1.65 ± 1.05	−0.40 ± 0.81
Afsharfar et al. (10)	Ma/Fe: 40 Intervention: 19, Control: 21	RA/DB/parallel	MI to MO	20–60	BDI	No	450 mg/day magnesium chloride	Placebo: starch	8	−3.53 ± 4.63	−0.67 ± 3.73
Nazarinasab et al. (5)	Ma/Fe: 60 Intervention: 30, Control: 30	RA/DB/parallel	Major	Intervention: 38 ± 9, Placebo: 39 ± 9	BDI	Different SSRIs	250 mg/day NR	Placebo	6	−19.24 ± 2.26	−16.77 ± 2.15
Rajizadeh et al. (15)	Ma/Fe: 53 Int: 26, Con: 27	RA/DB/parallel	MI to MO	20–60	BDI II	Some of the subjects	500 mg/day magnesium oxide	Placebo	8	−15.65 ± 8.92	−10.40 ± 7.90
Fard et al. (17)	Fe: 66 Intervention: 33, Control: 33	RA/TB/parallel	Major/PD	Intervention: 26 ± 5, Placebo: 27 ± 5	MDSS	No	320 mg/day magnesium sulfate	Placebo	8	−1.40 ± 1.73	−0.40 ± 1.34
Ryszewska-Pokraśniewicz et al. (11)	Ma/Fe: 37 Intervention: 17, Control: 20	RA/parallel	Major	Int: 48 ± 15, Placebo: 50 ± 12	HAM-D	Fluoxetine 20-40 mg/day	40 mg/day magnesium aspartate	Placebo	8	−19.24 ± 1.03	−17.10 ± 1.02
Mehdi et al. (16)	Ma/Fe: 12 Intervention: 6, Control: 6	RA/DB/crossover	MI to MO/TR	46 ± 9	HAM-D	No	4 g magnesium sulfate saluted in 5% dextrose infused in 4 h	Placebo: 5% dextrose infused in 4 h	1	−0.81 ± 1.03	−4.83 ± 1.01
	Ma/Fe: 12 Intervention: 6, Control: 6									−3.15 ± 1.00	−1.39 ± 1.00

RA, Randomized; TB, Triple-blinded; Ma, Male; Fe, Female; DB, Double-blinded; MI, Mild; MO, Moderate; PD, Postpartum depression; TR, Treatment-resistant; BDI, Beck’s depression inventory; MDSS, Mean depression symptom score; HAM-D, Hamilton depression rating scale; SSRI, Selective serotonin reuptake inhibitors; NR, Not reported. ^1^Values are mean ± SD or range (for age).

Only the study at Mehdi et al. (16) was a crossover trial, while all the other studies had a parallel design. With regard to the type of intervention, two studies administered magnesium sulfate (16, 17), two studies administered magnesium oxide (15, 18), one study administered magnesium chloride (10), one study used magnesium aspartate (11), and one study did not report the type of administered magnesium supplement (5).

In some studies, the baseline severity of depression was mild to moderate (10, 14–16), while in three studies, participants had major depressive disorders (5, 11, 17). With regard to Cochrane Risk of Bias Assessment Tool results, only two studies (16, 17) could be considered high-quality studies with a totally low risk of bias for all domains. Three RCTs (10, 14) had moderate quality, in which one part or more had an unclear risk of bias, and the other articles had low quality, having a high risk of bias for one domain or more (Supplementary Table 2).

### Findings from the meta-analysis

**Figure 2:**
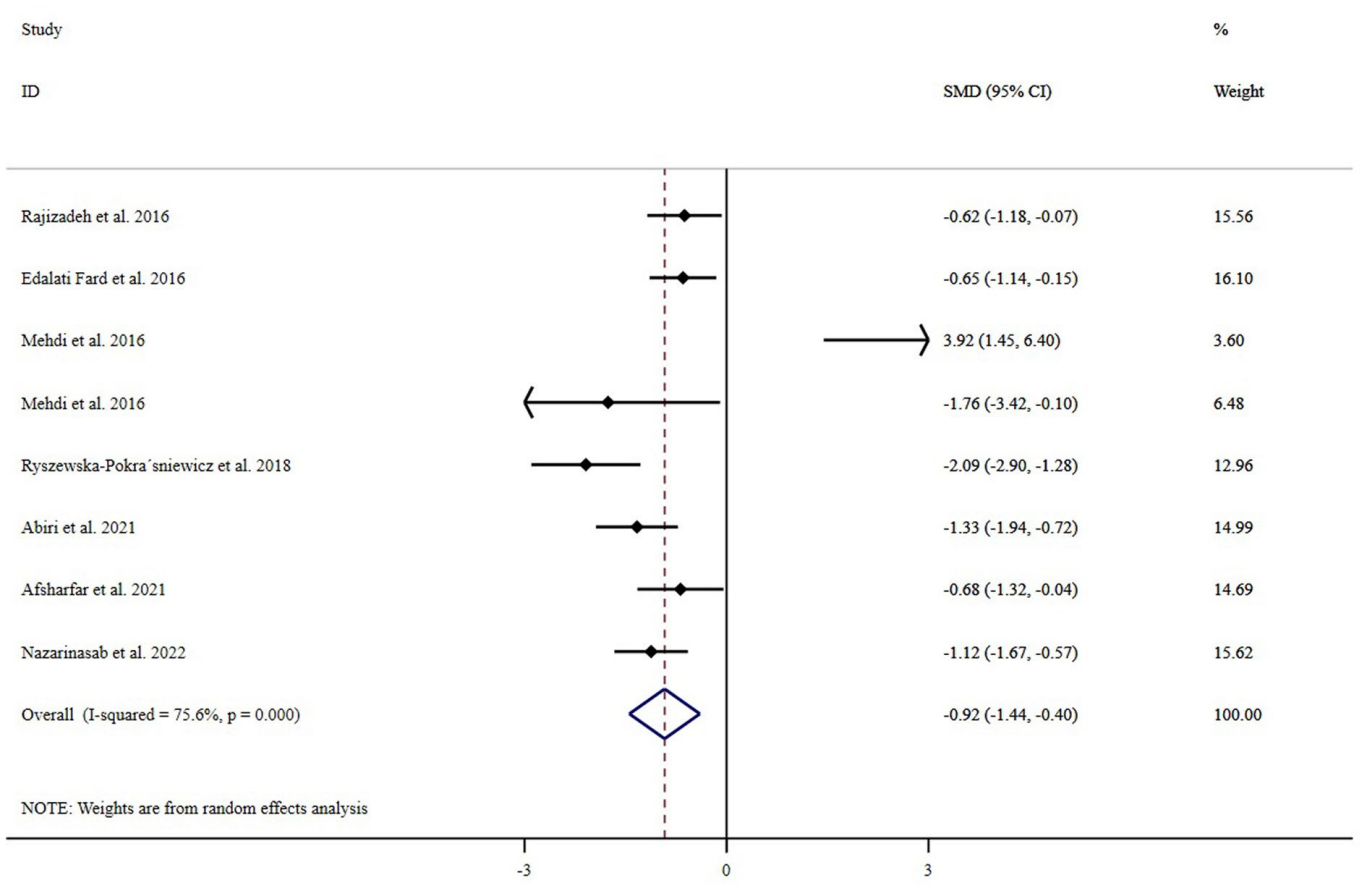
Effect of magnesium supplementation on depression forest plots, reported as standardized differences between intervention and control groups. Horizontal lines are regarded as 95% CIs. The diamond is viewed as pooled estimates from analysis (random effects). SMD, Standardized mean difference; CI, Confidence interval.

In total, seven randomized clinical trials with a sample size of 325 subjects were included in the analysis (5, 10, 11, 14–17). Combining these eight effect sizes from these studies indicated that magnesium supplementation causes a significant drop in depression scores compared with placebo [standardized mean difference (SMD): −0.919, 95% CI: −1.443 to −0.396, *p* = 0.001; Figure 2]. We also conducted a random-effect model analysis excluding the study from Mehdi et al. study as it used IV magnesium, and the result remained significant [standardized mean difference (SMD): −1.05, 95% CI: −1.38 to −0.71, *p* < 0.001]. Furthermore, we excluded studies by Ryszewska-Pokraśniewicz et al. (11) and Mehdi et al. (16) since they used the HAM-D test, which is a clinical-rated depression scale, and the random-effect analysis remained significant [standardized mean difference (SMD): −0.87, 95% CI: −1.145 to −0.590, *p* < 0.001]. However, between-study heterogeneity was significant (*I*^2^ = 75.6, *p* = 0.001). To detect potential sources of heterogeneity, subgroup analyses were performed (Table 2). We found that the type of depression assessment test and study location could explain this heterogeneity. All subgroup analyses showed reduced effects of magnesium on depression scores. Our subgroup analysis suggests that 250 mg/day or less of magnesium supplements may have a stronger effect than higher doses in reducing depression scores (Table 2). However, we found no significant association between studies that used BDI for depression assessment (95% CI: −1.231, 0.648) and those that used the infusion route for the intervention (95% CI: −1.376, 1.388).

**Table 2:** Subgroup analysis based on fixed effects models for the effect of magnesium supplementation on depression in adults aged ≥20 years.

	Effect size, *n*	SMD (95% CI)^1^	*p* within^2^	*I*^2^ (%)^3^	*p* heterogeneity^4^
Magnesium supplementation on depression					
Overall	8	−0.943 (−1.179, −0.706)	<0.001	75.6	<0.001
Intervention duration (week)					
<8	3	−0.968 (−1.475, −0.461)	<0.001	87.6	<0.001
8	5	−0.936 (−1.203, −0.668)	<0.001	68.0	0.014
Study location					
Iran	5	−0.864 (−1.115, −0.613)	<0.001	17.8	0.301
Non-Iran	3	−1.552 (−2.252, −0.853)	<0.001	90.2	<0.001
Assessment tool					
Beck’s depression inventory (BDI) I/II	4	−0.939 (−1.231, 0.648)	<0.001	22.4	0.276
Non-BDI	4	−0.949 (−1.353, −0.545)	<0.001	87.9	<0.001
Type of supplementation					
Oral	6	−0.971 (−1.211, −0.731)	<0.001	61.1	0.025
Infusion	2	0.006 (−1.376, 1.388)	0.993	92.8	<0.001
Magnesium dosage					
≤250 mg/day	3	−1.389 (−1.752, −1.026)	<0.001	47.8	0.147
>250 mg/day	5	−0.615 (−0.926, −0.304)	<0.001	72.9	0.005

Subgroup analyses for the effects of magnesium supplementation on depression in adults aged ≥20 years. SMD, Standardized mean difference; CI, Confidence interval. ^1^Obtained from the fixed-effects model. ^2^Refers to the mean (95% CI). ^3^Inconsistency, percentage of variation across studies due to heterogeneity. ^4^Obtained from the Q-test.

Excluding any single study did not affect the overall estimate for the effect of magnesium supplementation on depression status in the sensitivity analysis (range of summary estimates: −1.30, −0.59). Moreover, no evidence of substantial publication bias was found based on Egger’s test (*p* = 0.574).

## Discussion

The current meta-analysis showed a significant reduction in depression scores following magnesium supplementation in adults with depressive disorder.

Magnesium is crucial in modulating the central nervous system (CNS) (19). Depression is a common, debilitating, and potentially lethal disorder (2). The current systematic review and meta-analysis showed a significant effect of magnesium in reducing depression scores as measured by different instruments. In line with our study, in a review article by Serefko et al. (19), magnesium was considered effective in reducing depression. In one meta-analysis by Boyle et al. (20), magnesium was found effective in reducing anxiety. The study by Afsharfar et al. showed that the administration of 500 mg of magnesium per day can improve depression status in adults (10). Tarleton et al. (21) revealed that a 2-week intervention with 248 mg of elemental magnesium per day can lead to clinical improvement in patients with anxiety and depressive disorders. However, the study by Ryszewska-Pokraśniewicz et al. (11) failed to find significant improvement in depression scores in the magnesium plus fluoxetine group compared to fluoxetine alone. The included subjects had major depression, with 19 subjects having severe depression. This may explain why magnesium supplementation did not show a significant effect on depression reduction.

Our subgroup analysis showed no significant effect of magnesium supplementation on depression scores in studies that used the BDI for depression assessment. In addition, the reducing effect of magnesium on depression scores was more considerable among studies performed in Iran. It seems that cultural and economic differences between populations should be taken into account when investigating the effects of an intervention on depression symptoms. Moreover, different instruments should be used to assess depression because of substantial internal variations between the available methods. In addition, there was no significant effect in studies that used the infusion route for the intervention. It can be explained by the limited duration of intervention (mean 4 h) in those studies (16).

Studies indicate that magnesium deficiency contributes to the pathophysiology of mood disorders, suggesting an antidepressant effect of magnesium supplementation (19). Several pathways might play a role in these effects. Magnesium acts as a natural antagonist of calcium, blocks the NMDA receptor channel in a voltage-dependent manner, and prevents the flow of calcium ions through it. In addition, magnesium enhances the expression of the GluN2B subunit belonging to the NMDA receptor complex. Low magnesium levels in the hippocampus, plus high levels of both calcium and glutamate, may result in altered functioning of synapses in the human brain and lead to the development of mood disorders, including depression (22). Furthermore, magnesium intake has been associated with reduced systemic inflammation in the body (23). Systemic inflammation is a crucial risk factor for several psychological disorders (24). Finally, earlier studies have shown that magnesium intake has a role in normalizing sleep organization, and its deficiency is linked to some sleep disturbances (25). It should be noted that disorders of the sleep/wake cycle are related to the pathophysiology of depression (19).

In the current meta-analysis, we included all available evidence about the effect of all different types of supplemental magnesium compounds on depression. However, we would like to address some potential limitations when interpreting the findings. There was high heterogeneity between the studies included in the meta-analysis. We tried to explain such heterogeneity with different subgroup analyses. In the subgroup analysis, study location and depression assessment methods were found to be potential sources of between-study heterogeneity. In addition, differences in supplementation dosage, study duration, route of administration, and outcome assessment between included studies were other sources of bias, the influences of which we tried to reduce on our final results using several subgroup analyses, if possible. We were not able to find a safe margin for supplemental magnesium since no adverse effects were reported following magnesium supplementation in these studies. Moreover, some of the included studies involved patients with different conditions, such as postpartum depression (17) and treatment-resistant depression (16). Further studies about each type of disease should be addressed to reach a firm conclusion. All the studies we included had groups adjusted by age. However, some of them, such as Airi et al., did not adjust the groups by basement serum levels of magnesium and depression score, which might negatively affect our results (14). In addition to that, there were only two studies that used clinical-rated depression scales (11, 16). Finally, the study sample size of most included articles was small, and they built up a small sample size of 325 individuals in the current meta-analysis. More large studies are required.

In conclusion, the current meta-analysis showed a significant reduction in depression scores following magnesium supplementation. Further clinical trials are required to expand existing knowledge in this area.

## Data availability statement

The original contributions presented in the study are included in the article/Supplementary material, further inquiries can be directed to the corresponding author.

## Author contributions

MM: Writing – original draft, Writing – review & editing. MA: Writing – review & editing. SE: Writing – review & editing. AM: Writing – original draft, Writing – review & editing.
