# Supplementary material for: Magnesium supplementation beneficially affects depression in adults with depressive disorder: a systematic review and meta-analysis of randomized clinical trials
Source: Front Psychiatry. 2023 Dec 22;14:1333261. doi: 10.3389/fpsyt.2023.1333261 (PMC10783196; doi:10.3389/fpsyt.2023.1333261)
Supplement: Supplementary file 1 [file Table_1.DOCX]

**Supplemental Table 1**: The term list that was used to perform the systematic search on the effect of magnesium on depression

| **In Scopus, and Web of Science, PubMed** | | **n** |
| --- | --- | --- |
|  | 1. “Magnesium” |  |
|  | 2. “Mg” |  |
|  | 3. (1 OR 2) |  |
|  | 4. “major depression” |  |
|  | 5. “refractory depression” |  |
|  | 6. “depression scores” |  |
|  | 7. “affective disorders” |  |
|  | 8. “depressive disorder” |  |
|  | 9. “mental health” |  |
|  | 10. “depression” |  |
|  | 11. “Depressive Disorder, Major” |  |
|  | 12. (4 OR 5 OR 6 OR 7 OR 8 OR 9 OR 10 OR 11) |  |
|  | 13. "rct" |  |
|  | 14. "Randomized controlled trial" |  |
|  | 15. "Randomized clinical trial" |  |
|  | 16. "Random allocation" |  |
|  | 17. "Random assignment" |  |
|  | 18. trial |  |
|  | 19. trials |  |
|  | 20. randomized |  |
|  | 21. randomised |  |
|  | 22. controlled |  |
|  | 23. blind |  |
|  | 24. blinded |  |
|  | 25. crossover |  |
|  | 26. (13 OR 14 OR 15 OR 16 OR 17 OR 18 OR 19 OR 20 OR 21 OR 22 OR 23 OR 24 OR 25) |  |
|  | (3 AND 12) **In PubMed** | 1590 |
|  | (3 AND 12 AND 26) **In Scopus** | 1716 |
|  | (3 AND 12 AND 26) **In ISI Web of Science** | 494 |
|  | **Duplicate** | 783 |
| **In Google Scholar** | | 400 |
|  | Magnesium and depression  By searching the above combination in this engine, we screened the first 200 relevancy ranked papers to avoid missing any eligible studies. |  |
| **Total** | | 3417 |

| study | **Random sequence generation** | **Allocation concealment** | **Selective reporting** | **Blinding (participants and personnel)** | **Blinding (outcome assessment)** | **Incomplete outcome data** |
| --- | --- | --- | --- | --- | --- | --- |
| Abiri et al. 2021 | **L** | **L** | **L** | **L** | **U** | **L** |
| Afsharfar et al. 2021 | **L** | **L** | **L** | **L** | **U** | **L** |
| Nazarinasab et al. 2022 | **L** | **U** | **L** | **L** | **U** | **H** |
| Rajizadeh et al. 2016 | **L** | **L** | **L** | **L** | **U** | **H** |
| Edalatiard et al. 2016 | **L** | **L** | **L** | **L** | **L** | **L** |
| Tareton et al. 2017 | **L** | **H** | **L** | **H** | **H** | **L** |
| Ryszewska-Pokra´sniewicz et al. 2018 | **L** | **U** | **L** | **L** | **L** | **L** |
| Mehdi et al. 2016 | **L** | **L** | **L** | **L** | **L** | **L** |

**Supplemental Table 2**: Results of risk of bias assessment for randomized clinical trials included in the current meta-analysis on the effects of magnesium supplementation on depression^1^

^1^Each study was assessed for risk of bias using the Cochrane Risk of Bias Assessment tool (9). Each domain was given a “high risk” score if the study comprised methodological defects, and a “low risk” if it did not have any methodological flay for that item, and an “unclear risk” score if the there was not sufficient information to establish the risk. We considered overall risk of bias following these criteria: (1) Low; if all the domains were marked as “low risk”, (2) Moderate; if one or more domains were marked as “unclear risk”, and (3) High; if one or more domains were marked as “high risk”..
